# Accelerating Faceting Wide-Field Imaging Algorithm with FPGA for SKA Radio Telescope as a Vast Sensor Array †

**DOI:** 10.3390/s20154070

**Published:** 2020-07-22

**Authors:** Yuefeng Song, Yongxin Zhu, Tianhao Nan, Junjie Hou, Sen Du, Shijin Song

**Affiliations:** 1 School of Microelectronics, Shanghai Jiao Tong University, Shanghai 200240, China; songyuefeng@sjtu.edu.cn (Y.S.); nanth1996@sjtu.edu.cn (T.N.); hjj123@sjtu.edu.cn (J.H.); du_sen@sjtu.edu.cn (S.D.); jennifer-song@sjtu.edu.cn (S.S.); 2 Shanghai Advanced Research Institute, Chinese Academy of Sciences, Shanghai 201210, China; 3 University of Chinese Academy of Sciences, Beijing 100049, China

**Keywords:** SKA, FPGA, cloud computing, big data technologies, phase rotation, gridding

## Abstract

The SKA (Square Kilometer Array) radio telescope will become the most sensitive telescope by correlating a huge number of antenna nodes to form a vast array of sensors in a region over one hundred kilometers. Faceting, the wide-field imaging algorithm, is a novel approach towards solving image construction from sensing data where earth surface curves cannot be ignored. However, the traditional processor of cloud computing, even if the most sophisticated supercomputer is used, cannot meet the extremely high computation performance requirement. In this paper, we propose the design and implementation of high-efficiency FPGA (Field Programmable Gate Array) -based hardware acceleration of the key algorithm, faceting in SKA by focusing on phase rotation and gridding, which are the most time-consuming phases in the faceting algorithm. Through the analysis of algorithm behavior and bottleneck, we design and optimize the memory architecture and computing logic of the FPGA-based accelerator. The simulation and tests on FPGA are done to confirm the acceleration result of our design and it is shown that the acceleration performance we achieved on phase rotation is 20× the result of the previous work. We then further designed and optimized an efficient microstructure of loop unrolling and pipeline for the gridding accelerator, and the designed system simulation was done to confirm the performance of our structure. The result shows that the acceleration ratio is 5.48 compared to the result tested on software in gridding parts. Hence, our approach enables efficient acceleration of the faceting algorithm on FPGAs with high performance to meet the computational constraints of SKA as a representative vast sensor array.

## 1. Introduction

Remarkable advances in sensor technology and acceleration of Internet of Things (IoT) technology have led to explosive increases in the volume and rate of sensing data, which poses mounting challenges to data storage and real-time processing in the cloud-based system. With the evolution of radio astronomical observation technology, radio telescopes suffer from similar challenges to exclusive giant sensor arrays.

The Square Kilometer Array (SKA) is a multinational astronomical project designed to build the next generation radio telescope to operate over a wide wavelength range of meters to centimeters. It will have an unprecedented large collection area of approximately one square kilometer with a maximum baseline of 3000 kilometers, providing full detection sensitivity for frequencies up to at least 14 GHz, which is 50 times higher than the Karl G. Jansky Very Large Array (VLA). SKA features multiple field-of-view (FoV) of more than one square degree at higher frequencies to achieve large sky coverage [1].

The SKA will be built in two phases: SKA1 and SKA2, to provide continuous frequency coverage from 50 MHz to 14 GHz, due to the size of the project. SKA1 consists of two instruments: SKA1-low and SKA1-mid. SKA1-low is an aperture array instrument grouping 1024 stations, each containing 256 dual-polarized antennas to cover a total collecting area of 0.4 km^2^, which will receive signals ranging from 50 to 350 MHz. SKA-mid is constructed to collect an area of 33,000 m^2^ by using 200 single-pixel feed dishes, including 64 MeerKAT dishes, capable of receiving signals between 350 MHz and 14 GHz [2,3]. The total collecting area of the SKA will exceed one square kilometer, which makes the SKA the largest synthesis radio telescope ever built, by some margin.

The SKA’s unprecedented ultra-large-scale data will bring big challenges to the SKA data transporting, storing, analyzing, processing, and interpreting. Statistically, an exabyte of raw data is generated by an array of antennas one day, which makes the SKA scientific data processing (SDP) the bottleneck of the whole data flow [4]. The SKA will push astronomy into exascale signal processing and computing in the next decade. We will need to be clever with both new signal processing algorithms and practical implementations of those algorithms, in order to sensibly deal with the amount of data processed.

As the wide-field imaging algorithm in the SKA project, faceting is an excellent algorithm approach towards solving image construction from sensing data where earth surface curves cannot be ignored [5]. The phase rotation and gridding are the most time-consuming phases in the faceting procedures. However, the traditional processor of cloud computing, even if the most sophisticated supercomputer s used, cannot meet the extremely high computation performance requirement [6].

The accelerators based on heterogeneous hardware is typically selected as a scalable circuit to offer much higher performance than CPU (Central Processing Unit) -based generic computing architectures [7]. Taking the flexibility and cost of prototype design into consideration, we decided to use FPGA (Field Programmable Gate Array) to implement the prototype of scientific data processing algorithm without the precedent experience of ASIC (Application-specific integrated circuit) -based prototype design. In this paper, we explore the FPGA-based acceleration of key procedures of faceting, which is one of the most computationally demanding procedures in SKA1-SDP (Science Data Processor) [8].

The contributions of this paper can be summarized as: (1) We proposed an efficient parallelization and pipeline structure designed for the two most time-consuming procedures of the faceting algorithm and implemented it on the FPGA, (2) we further improved the computing performance of the phase rotation accelerator through the optimized pipelined computing kernel, and (3) we presented a comprehensive analysis of the achieved performance.

This paper is an extension of Reference [9]. Compared to previous work, we include a new hardware architecture for the phase rotation implementation on the FPGA and take the data transmission between the host and extended hardware into consideration in Section 5.4. We add comprehensive literature references, and some additional publications are discussed in Section 3. We include the faceting algorithm bottlenecks analysis in Section 4. In the section of the evaluation, the results of the optimized approach to acceleration are provided and more previous implementations are compared.

The remainder of the paper is organized as follows. Section 2 introduces the background, Section 3 introduces related works, Section 4 describes the faceting algorithm behavior and makes a bottlenecks analysis, and Section 5 illustrates the scheme of the phase rotation acceleration on the FPGA. Section 6 demonstrates the implementation approach of the FPGA-based gridder, Section 7 shows the experimental results and analyzes them, and we draw a conclusion and present the future work in Section 8.

## 2. Background

Radio telescopes detect electromagnetic waves from cosmic radio sources. The signals are used to create a map of the sky, including the location, intensity, and polarization of the light source. Unlike optical telescopes, which typically consist of a single receiver, modern radio telescopes such as SKA consist of many small antennas. Three steps are required in general to build a sky image: (1) different correlated pairs of stations generate digital signals, namely visibilities, (2) instrument parameters and environmental effects are estimated by calibration, and (3) the imaging steps partially correct the visibility into the sky image [10].

The low-frequency radio telescope arrays have the common characteristics of the large FoV, high dynamic range, and high sensitivity. The geometric structure of the array is non-coplanar, and the baseline in the array is non-coplanar with the equatorial plane of the earth. Therefore, under the condition of wide FoV in the low frequency, the traditional two-dimensional (2D) Fourier transform approximation is no longer true [11]. On the contrary, an imaging algorithm in the process of inversion is required to consider w-term, which describes the error caused by the non-coplanar array [12]. Decrease of the quality of the image will be amplified with the increasement of the observation FoV of the radio interference array, if w-term is ignored. Thus, it is extremely import to solve the problem in the low-frequency radio telescope array imaging.

At present, there are several kinds of treatment w-terms of imaging methods: Faceting [13], three-dimensional (3D) Fourier transform [12], w-projection [14], w-stacking [15], and warped snapshots [12]. In addition, hybrid methods such as w-stacking [16] are also very practical, which can overcome the defects of the original methods and inherit their advantages. Although the 3D Fourier transform method is the simplest, in theory, it has the lowest efficiency and the largest amount of computation, and it is rarely used in practical applications [12]. The Common Astronomy Software Applications (CASA) have effectively implemented faceting and w-projection algorithms. However, traditional computing platforms cannot meet the required performance of processing by these algorithms.

As the largest aperture radio telescope throughout history, the SKA will produce a large volume of data for processing. The MWA (Murchison Widefield Array) is the SKA pilot project which has accumulated 24 petabyte (PB) scientific data after a 4-year operation. As the largest radio telescope at the lowest frequencies, LOFAR (Low-Frequency Array) will produce 6 PB high spatial resolution image every year as well and the data rate of ASKAP (Australian Square Kilometer Array Pathfinder) that was built in 2014 will reach 72 Tbps [17]. However, the data scale of these projects is much less than that of SKA. Only the SKA1′s raw data rate reaches 160 Tbps statistically [18], but the antenna amount of SKA1 is only 10% of SKA2, which means the data rate will expand to 100 times by the time of SKA2. Faced with such a magnitude of data, it is practically impossible for a system to store and further process them. Consequently, a real-time processing system is necessary in case the data flow pipeline is blocked for the ultra-scale data of SKA.

The real-time processing of such large-scale data poses a great challenge to the performance of modern computers. The computing demand of SKA1 is estimated to reach a magnitude of 100 PFlop/s (Peta Floating Point Operations per Second). Even if the amount of antenna is reduced by a compressor and the computing efficiency is increased by 10%, the peak performance demand still reaches 300 PFlop/s [19], which means that only a supercomputer can satisfy the requirement strictly. However, the peak performance that the best supercomputer Summit can reach is 200.795 PFlop/s [20], with insufficient computing power to meet the requirement. Hence, SKA scientific data processing is a computationally and data-intensive application. Since the constraint of data I/O rate, the insufficient power of the traditional HPC (high-performance computation) forces more advanced computing architecture designed to handle the sophisticated scientific data processing challenge in the SKA project.

Cloud computing is a platform with excellent computing services, including the ability to scale elastically, and it can be used to process the huge amount of data in the SKA project [21]. Traditional cloud computing service providers build large data-centers with a huge number of interdependent commodity computers with CPU as the key computing unit to handle the ever-growing challenge on performance. Emerging heterogeneous cloud computing as a special type of the parallel computing manner is flexible to assign different forms of computing tasks according to algorithm structural characteristics. The heterogeneous computing structure based on CPU + FPGA is mainly used to solve the main problems of high performance, high efficiency, and big data processing, which show the great potential of accelerating the SKA’s algorithm [22], as FPGA is good at parallel designing and combines the advantages of parallel processing ability and low energy consumption [23]. Therefore, this paper focuses on the faceting imaging algorithm accelerator implemented on the FPGA to realize the key phases computing in the SKA’s data processing.

## 3. Related Works

Some recent works focus on the data processing of low-frequency synthetic-aperture interferometer arrays and demonstrate the optimization performance of the wide FoV imaging. Humphreys and Cornwell [24] provide an analysis of the convolutional resampling algorithm in gridding concerning the radio-astronomy imaging pipelines of the ASKAP and describe a GPU (Graphics Processing Unit) gridder optimized to perform real-time imaging with maximum hardware memory bandwidth utilized. Romein [25] presents a new efficient strategy to assign the work of gridding distributed over the threads and thread blocks for w-projection on GPUs. The strategy efficiently convolves and grids the visibility data to minimized device-memory accesses, without relying on sorting or searching visibilities. They also compare the performance on different high-end platforms in CUDA (Compute Unified Device Architecture) and OpenCL (Open Computing Language) to further measure the performance. Based on Romein’s algorithm, Merry [26] presents a thread coarsening method where the multiple work items are merged into one across parallel work items to improve instruction-level parallelism and the efficiency of gridding computing for single-polarization and quad-polarization on the target GPUs. Veenboer et al. [10] initiated the CPUs-based and GPUs-based image-domain gridding (IDG) algorithm and first presented the efficient degridding implementation on GPUs. They presented a parallelization and optimization strategy on gridder kernel and degridder kernel and the result was demonstrated in close to optimal performance on different platforms of CPUs and GPUs. Lao et al. [27] proposed a faceting imaging algorithm based on the MPI (Message Passing Interface) + OpenMP (Open Multi-Processing) and a faceting imaging algorithm based on the MPI + CUDA (Compute Unified Device Architecture). The verification result indicates that the MPI + CUDA method outperforms the MPI + OpenMP method concerning correctness ratio and throughput.

These works all concentrate on hardware accelerators of imaging approaches based on CPU or GPU. Veenboer and Romein [28] implemented and optimized a radio-astronomical imaging application on a target FPGA. They compare architectures, programming models, optimizations, performance, energy efficiency, and programming effort to highly optimized GPU and CPU implementations. The result reveals that although theoretical peak-performance for these devices is almost identical, the FPGA and GPU perform much better than the CPU and they consume significantly less power. In absolute terms, the GPU is the fastest and most energy-efficient device. The results also show that the FPGA resource usage can be efficiently optimized, but that optimizing for high clock speed is difficult.

On top of the exploration for performance optimization of imaging accelerators, some previous work concentrates on the evaluation method to resource allocation robustness in multi-core systems and job scheduling mechanisms for parallel processing in the cloud system. Li et al. [29] present a metric within a systematic method to measure the impact of inaccurate information on random resource allocation and evaluate how inaccurate probability arguments may affect the performance by providing the model for information inaccuracies. Besides, they provide a comparison of several greedy heuristics utilizing incorrect information. Li et al. [30] develop a preemptable task scheduling mechanism in cloud systems. Besides, they propose feedback dynamic list scheduling and feedback dynamic min-min scheduling as feedback scheduling algorithms for the dynamic scheduling mechanism to settle the invisibility for tasks received in different schedulers. They also introduced a feedback program when scheduling jobs to minimize the effect of competition on resources.

In the actual application of astronomical observation, not only the data throughput but the power consumption has to be considered. The FPGA features high performance, low energy consumption, and programmable hardware without instruction fetching and instruction decoding, which exist in the procedures of data processing based on CPU or GPU. In the Intel CPU, decoding alone accounts for 50% of the energy consumption of the whole chip due to the CISC architecture. The fetching and decoding also consumes 10 to 20 percent of the power [31]. Designed as a SKA precursor, MeerKAT is located in the central Karoo in South Africa, where less than 5 MW of power is required due to energy abundance. Besides, as the advanced compilation techniques rapidly develop, high-level synthesis facilitates an algorithm mapped to FPGA for scientists to achieve the desired algorithm acceleration results.

Although GPU supports a higher precision floating-point, it is not suitable for low-power and high-throughput application scenarios. In astronomical observations, the input data is converted into a limited number of data bits by means of analog-to-digital conversion. For example, in the observation of pulsars with FAST (Five-hundred-meter Aperture Spherical radio Telescope), the precision of data less than 8 bits with data clipping is usually sufficient for subsequent processing and analysis.

To the best of our knowledge, there are no studies of FPGA-based phase rotation combined with gridding of facet accelerators for FoV imaging, except for our previous work [9] on the FPGA-based accelerator with adequate performance for the imaging application. Besides, the related work of the imaging accelerators concerns the computing delay of each transaction, and the issue on the large volume of data is not taken into consideration for astronomical imaging acceleration. In this work, we focus on accelerating the data processing of the two most time-consuming algorithms and further research on computing architecture to optimize the performance.

## 4. Faceting Algorithm Behavior and Bottlenecks Analysis

Low-frequency radio telescope array is a giant sensor array that has the common characteristic of high sensitivity, large FoV, and high dynamic range. However, the array is facing various difficult issues of which the most critical one is the non-coplanar baseline effect.

Due to the non-coplanar geometry of the array, the earth’s equatorial plane and non-earth baseline form a non-coplanar plan. The phase of w-term from w direction will be far larger than 1 during the large-field observation period. In the view of low-frequency and large FoV, traditional two-dimensional Fourier Transform approximation does not exist [11]. Instead, the imaging algorithm is required to consider w-term, which involves the error caused by non-coplanar array [32].

Imaging quality decrease caused by neglecting w-term will be amplified with the width increase of radio interference array FoV. Therefore, the issue must be solved for the low-frequency radio telescope array. The wide FoV imaging is one of the essential points in the data processing of low-frequency synthetic-aperture interferometer arrays for the SKA. At present, all the algorithms of w-snapshot, w-projection, and w-stack are used to correct the image of the whole FoV promptly. The results show that the optimized algorithm can only effectively reconstruct the sky image with low resolution [33].

**Figure 1:**
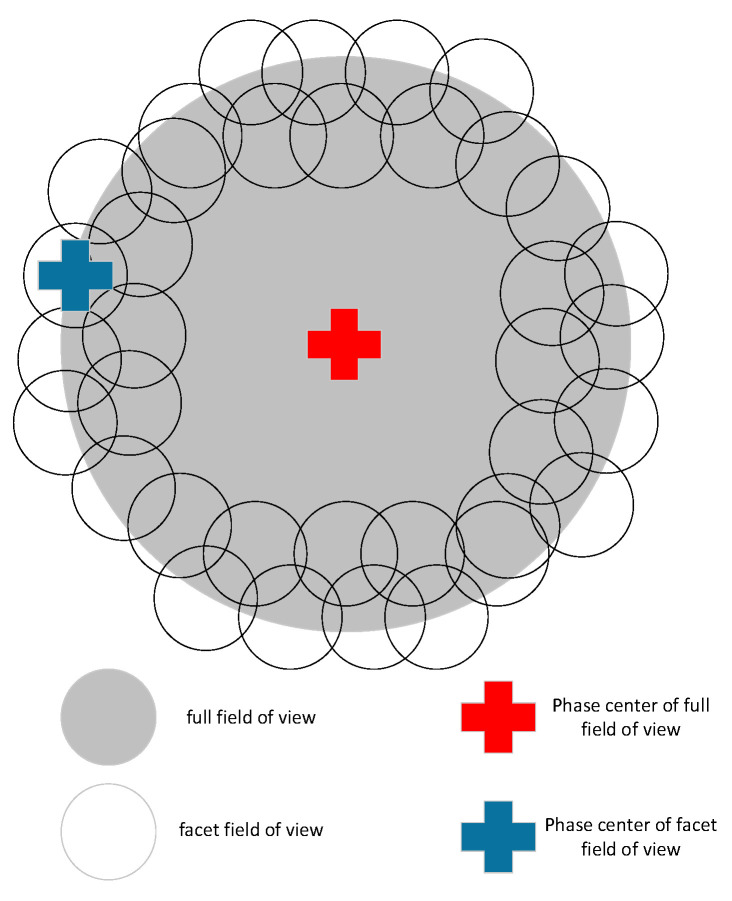
The principle of facet split for facet imaging.

Faceting is a typically used wide-field imaging algorithm which overcomes the shortcomings of original methods and inherits their advantages [13]. Compared with these approaches, the FoV in the faceting procedures is divided into multiple facets, with each of them devoted to the traditional two-dimensional transformation method to rebuild the sky image, and the views of all facets are stitched together. As is shown in Figure 1 [34], a wide FoV is divided into a puzzle consisting of several small facet FoVs, on which the 2D Fourier Transform approximation is valid, and each facet has its phase center.

Faceting highly enhances the scalability to suit the project where the image of ten billion pixels is produced for the reconstruction of a sky image with a large number of pixels. For the reconstruction of the large pixel sky image, it has better extensibility and suits the project, which may give rise to an image of ten billion pixels, such as the SKA. In addition, compared with other algorithms, another advantage in the faceting approach is that it can directly make corrections on the data for the direction-dependent effect before gridding and imaging [35]. Hence, this paper will make a parallel optimization and reformation on the faceting algorithm.

The FoV of each facet is small in size, which provides convenience for image procedures by using the two-dimensional Fourier transformation method.

**Figure 2:**
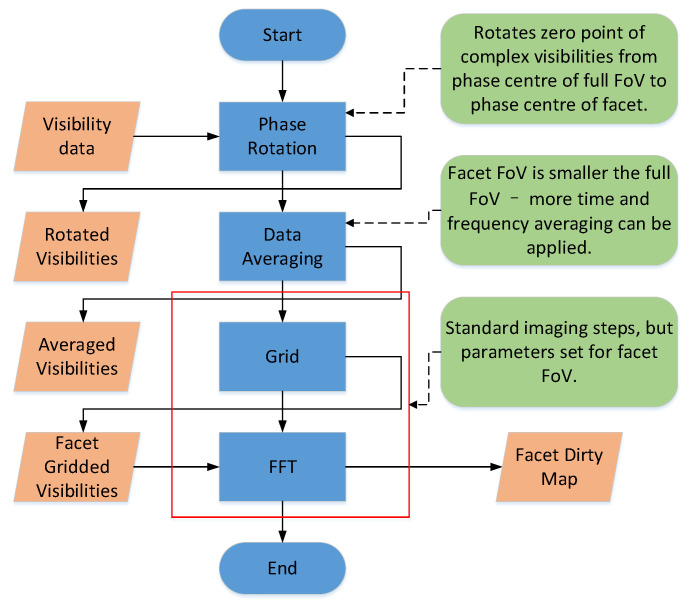
The procedure of Facet Imaging on one facet.

The detailed imaging process for each facet FoV is shown in Figure 2 [34]. Firstly, the original visibility data alters their phase center during phase rotation. Due to the facet FoV being much smaller than the full FoV, the data for each facet is to average the time and frequency before rasterization. 

The smearing effects of facets range from distinct FoV radii and include time average and bandwidth smearing effects. The time-average smearing effect is substantially the axial blur of the image caused by the rotation of the earth and the bandwidth smearing effect is the radial image blur caused by the chromatic aberration effect. Finally, the conventional imaging process is applied to acquire a dirty image. The advantages of the faceting imaging algorithm are that the field requires almost no consideration of the w-term and the small FoV allows for gridding and FFT for imaging.

We use SkyCoord objects in the software to represent an ICRS (International Celestial Reference System) sky position which consists of RA (Right ascension) and Dec (Declination). Given the software running result of 95,865 visibility samples, on one facet, of shifting phase-center from image phase center <SkyCoord (ICRS): (RA, Dec) in degee (8.1917712, −47.5582547)> to visibility phase center <SkyCoord (ICRS): (RA, Dec) in degee (15, −45)>, shown in Table 1, the most time-consuming part of facet imaging is phase rotation and gridding. Therefore, the acceleration of phase rotation and gridding is necessary and will be discussed in the following parts.

## 5. Phase Rotation Acceleration

In this section, we discuss implementation details of the phase rotation algorithm on FPGA and show how accelerator engines are integrated into hardware architecture to realize a complete system theoretically.

The ARL (Algorithm Reference Library) [36] is designed by the SKA scientific data processor to present calibration and imaging algorithms in a simple Python-based form for aperture synthesis imaging based on Numpy. The core procedure of the phase rotation published in ARL is expressed as the following formula.

(1) $$V\left(u,v,w\right)=V\left(u,v,w\right)*E^{-2\pi j\left(ul+vm+w\left(\sqrt{1-l^{2}-m^{2}}-1\right)\right)}$$

### 5.1. Calculation Procedures Analysis

As is shown in the Formula (1), the visibility is derived from the original visibility multiplying a phase rotation factor $E^{-2\pi j\left(ul+vm+w\left(\sqrt{1-l^{2}-m^{2}}-1\right)\right)},$ which is defined as a phasor. Different variables in one group comprised of l and m represent different directions of a facet. It means the value of l, m, and relation $\left(\sqrt{1-l^{2}-m^{2}}-1\right)$ is fixed on each facet. According to the result of software running, there are 95,865 visibility data on one facet, which means 95,865 phasors are required to be calculated. For one phasor, it needs to fetch a set of data including three-dimensional coordinates of u, v, and w, then time, with 2 πl, 2 πm, and $2\pi \left(\sqrt{1-l^{2}-m^{2}}-1\right)$. Then, the imaginary exponent with the previous result is calculated. Therefore, the computation magnitude is obviously huge and consumes a lot of time practically.

### 5.2. Preliminary System Design

The system design starts with visibility phase rotation implementation on one facet. The parameter pair of l and m represents the horizontal direction cosine and orthogonal direction sine respectively, involved in the phase tracking center. The input of the phase rotation system includes complex single-precision floating-point visibility data and corresponding coordinate (u, v, w) with each single-precision floating-point of each item. The data magnitude of each facet imaging performed is shown in Table 2.

Due to the 1.1 MB size of data, a total less than a piece of Block RAM (BRAM) of FPGA, all the data can be stored in it. Besides, there is scarcely any delay during data reading and writing which benefits from the high access speed of Block RAM to reduce the design difficulties.

To perform the imaginary exponent calculation, Euler formula is a typical approach to transfer the imaginary exponent into triangle function, which is conducive to further processing:

(2) $$e^{j\cdot \theta }=cos\theta +j\cdot sin\theta$$

Then, the original formula of phasor can be expressed as:

(3) $$\mathrm{Phasor}=e^{-2\pi j\left(ul+vm+w\left(\sqrt{1-l^{2}-m^{2}}-1\right)\right)}\;=\cos[2\pi \left(ul+vm+w\left(\sqrt{1-l^{2}-m^{2}}-1\right)\right]-j\cdot sin[2\pi \left(ul+vm+w\left(\sqrt{1-l^{2}-m^{2}}-1\right)\right]$$

The imaginary exponent shown in Formula (3) is transformed into the calculation of floating-point multiplication and triangle function, which is easier to be processed on the FPGA.

**Figure 3:**
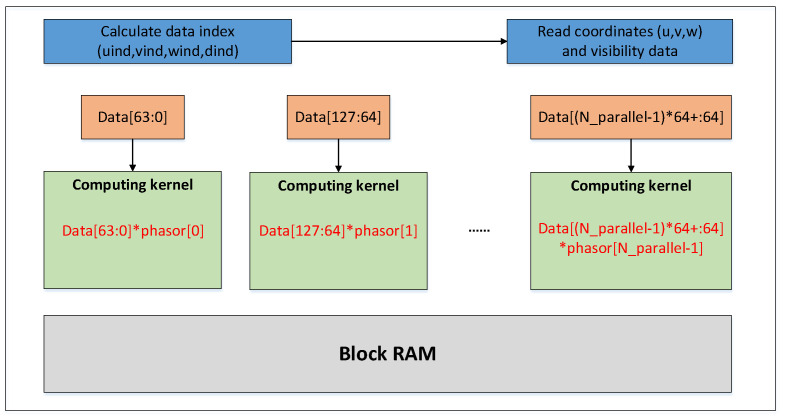
Parallel process flow of phase rotation algorithm.

Due to the independence of corresponding coordinates and visibility with each other in a facet, parallel processing is feasible for them without collisions among the data. Another feature of this formula is the complex processing procedures. Therefore, it is crucial to design an appropriate pipeline for data flow processing in high efficiency and accelerate the calculation of the formula. The flow of processing in parallel is shown in Figure 3.

### 5.3. Key Parts of the Design

After the overall structure of the system is determined, it needs to be further refined to complete the design of some key components.

#### 5.3.1. Data Reading and Writing

As is seen from the previous analysis, four reads and one write are needed for each phase rotation of visibility data. It is commonly known that I/O latency generated when data reading and writing may hinder the peak performance, which concerns the operation rate of the system. Consequently, it is necessary to design an advanced module for a less time-consuming design to make the system more efficient. Block RAM is an efficient memory with no delay and the coordinate and the visibility data are stored in Block RAM in the file format of Xilinx Coefficient (.COE) through BRAM IP (Intellectual Property) core initialized. Since the data reading and writing is fixed in width, data with the reading and writing bit width is required to be aligned.

#### 5.3.2. Floating-Point Operation Unit

The procedure of phase rotation contains multiple floating-point addition and multiplication. Therefore, it is necessary to decrease the cost of floating-point calculation. Xilinx FPGA officially provided IP core for floating-point addition and multiplication which outperforms the manually built adder or multiplier from performance aspects. The floating-point IP core is then chosen to improve the clock frequency, improve the calculation accuracy, reduce the critical path length, and eliminate the disparity in the calculation results due to insufficient precision of floating-point operations.

In view of complex imaginary number addition and multiplication, there is no provided IP core. So, preprocessing is required so that the complex is divided into a real part and an imaginary part, with multiplication and addition performed separately.

Complex addition:

(4) $$(x_{1}+j\cdot y_{1})+\left(x_{2}+j\cdot y_{2}\right)=\left(x_{1}+x_{2}\right)+j\cdot \left(y_{1}+y_{2}\right)$$

Complex multiplication:

(5) $$(x_{1}+j\cdot y_{1})*\left(x_{2}+j\cdot y_{2}\right)=\left(x_{1}\cdot x_{2}-y_{1}\cdot y_{2}\right)+j\cdot \left(x_{2}\cdot y_{1}+x_{1}\cdot y_{2}\right)$$

**Figure 4:**
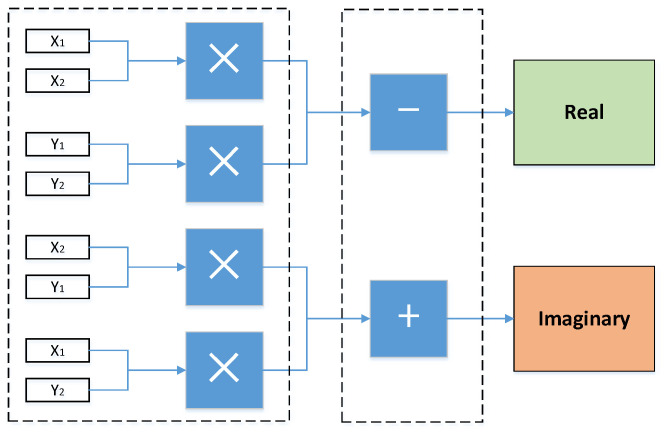
Complex adder and multiplier.

Four multipliers, one adder, and one subtractor are built on demand to accomplish a complex multiplication overall. The structure is shown in Figure 4. The adder and subtractor’s latency are 11 clock periods. The multiplier’s latency is set as 8 clock periods.

#### 5.3.3. Triangle Function

After the imaginary exponent is transferred into a triangle function, which is a time-consuming part during the computation, it is necessary to decrease the latency of the triangle function.

To reduce resource usage for the triangle, we investigated how lookup tables can be used as an alternative to the compiler-generated version and customized the Cordic IP core that Xilinx FPGA provides to calculate the triangle function with lower latency and high precision, since the Cordic (Coordinate Rotation Digital Computer) algorithm is a common method to calculate trigonometric, hyperbolic, and other mathematical functions. It is a digital algorithm that generates one output digit per iteration [37]. This allows us to tune the accuracy of the algorithm to the application requirements. Additional iterations produce a more precise output result. Accuracy is another common design evaluation metric alongside performance and resource usage. Cordic performs simple computations using only addition, subtraction, bit shifting, and table lookups, which are efficient to implement in FPGAs and more generally in hardware [38].

**Figure 5:**
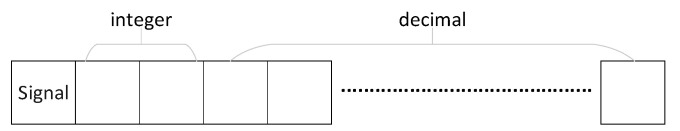
Input phase format of Cordic IP core.

The output data is cosθ and sinθ derived from the phase θ as an input. The input and output format of the Cordic IP core is characterized by fixed-point data that floating-point data cannot be delivered into the computing module directly. Thus, it is necessary for floating-point to be transformed into fixed-point. Besides, a conversion module from fixed-point to floating-point data format on the contrary is also needed. In view of the non-standardized transformation, the data signal and the integer width are three, and any remaining bits are used for the fractional portion of the number, as Figure 5 shows.

**Figure 6:**
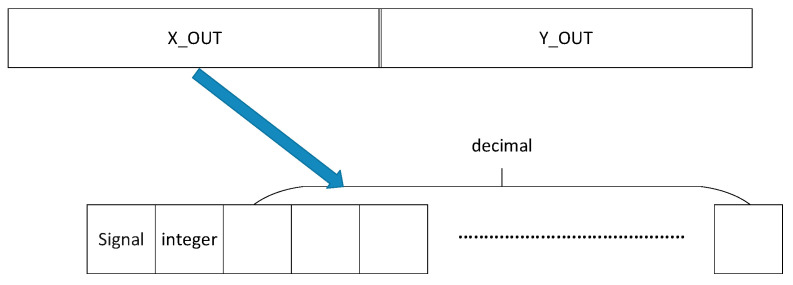
Output format of Cordic IP core.

The output contains two items: X_OUT and Y_OUT, which represents cosθ and sinθ, respectively (assume that the input phase is θ). Each output part is a fixed-point with a one-bit signal, one-bit integer, and the remaining bits representing the decimal, as it shows in Figure 6.

However, due to the constant single-precision floating-point as the data flow format in the pipeline, a converter is forced to transform the data in floating-point format into that in fixed-point format as the intermediate form for Cordic input on demand. Conversely, a converter is also needed to transform the output data into that in floating-point format.

#### 5.3.4. Pipeline Structure

Given the fact that the operating rate of hardware acceleration devices closely depends on the frequency of the clock which drives the processor core, it is required for the computing platform to embed the pipeline pattern inside of the processing procedures among which the integrated task is broken up into smaller parts intending to compress the size of the critical path and to boost the system clock frequency.

The processing steps consistent with timing sequence are detailed as follows:

1. Import coordinate data of u, v, and w into the kernel from on-chip memory.
2. Calculate input phase θ by $2\pi (ul+vm+w\left(\sqrt{1-l^{2}-m^{2}}-1\right)$.
3. Calculate core trigonometric function by cosθ and sinθ.
4. Import a piece of visibility data from the on-chip memory.
5. Calculate the sum of the product with visibility and (cosθ+j·sinθ)
6. Output calculation result back to the on-chip memory.

**Figure 7:**
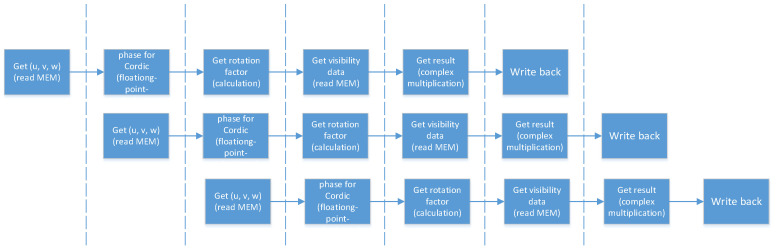
The pipeline of the phase rotation acceleration system.

Without hardware conflict or data conflict in the operations above, procedures can be conveniently integrated into a pipeline. The pipeline of the phase rotation acceleration system is shown in Figure 7.

### 5.4. Hardware Architecture Design

In this section, we discuss implementation details of the phase rotation algorithm on FPGA and show two approaches of accelerator design and how accelerator engines are integrated into hardware architecture to realize a complete system theoretically.

#### 5.4.1. Previous Work on the Architecture Design

In the previous work on the architecture design, the computing kernel on the FPGA is designed for the computing part of the algorithm. Due to incomplete pipeline optimization of the computing kernel, the iteration interval of a single computing kernel was 40 clock cycles, which were assumed as the cycle of the data acquisition from BRAM. To make full use of on-chip resources, we combined the two kernels into a single computing engine, so that each input cycle will read two groups of data (U_in, V_in, W_in, Data_in), of which Data_in is the visibility data, and one set of coordinates and visibility data can be processed every 20 clock cycles.

**Figure 8:**
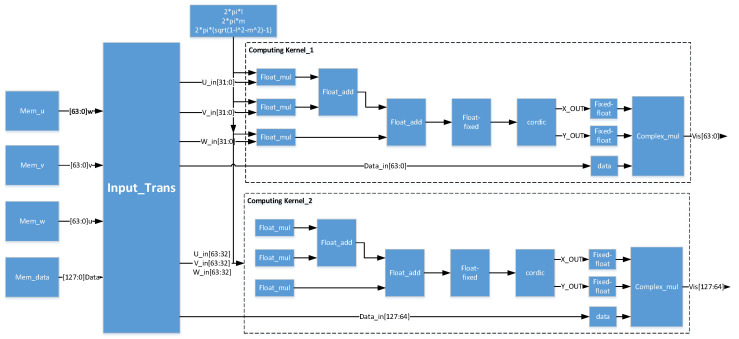
The overall structure of phase rotation algorithm designed in DKs.

We call such an acceleration system with a double parallel process structure a Duel Kernels structure (DKs), and it is shown in Figure 8. Two groups of data combined are sent into the system, then divided into a separated pipeline and processed in one cycle.

#### 5.4.2. Optimized Architecture Design

The DKs design is only for the datasets of finite volume. The system periodically obtains data from the .COE file in BRAM after initialization. The system operation under the large scale of data is not considered. The design of this paper innovatively takes the relationship between the host part based on CPU and the hardware accelerator based on FPGA into consideration, and simulates the transmission and caching process of real data from CPU to FPGA in view of the actual physical bandwidth limitation. The upgraded design of this paper also fully optimized the pipeline of the computing kernel to make the task interval reduce to 1 clock.

**Figure 9:**
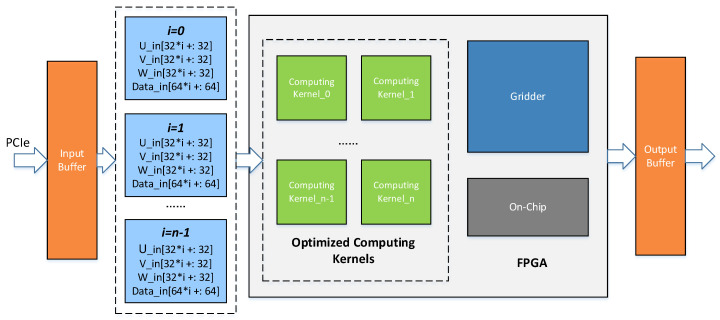
Overall optimized structure of the phase rotation algorithm.

The overall optimized FPGA-based hardware architecture of the phase rotation algorithm is shown in Figure 9. The data transformation wrapped as transactions to be processed between master memory on the host and global memory beside the FPGA kernel typically occurs across a peripheral high-speed serial interface. The PCIe (peripheral component interconnect express) -based heterogeneous runtime interactions require data delivered efficiently by using the direct memory access (DMA) to reduce the occupancy in the resources of the CPU [39]. The interactions with the accelerator managed by the data flow framework are restricted by bandwidth limitation contributing to non-full-speed acceleration, even though a high throughput processing kernel is deployed on the FPGA side. In the optimized FPGA-based accelerator design, a high bandwidth transceiver supporting PCIe is applied to diminish the bottleneck for data flow to running in maximum volume.

To demonstrate the feasibility of the FPGA-based phase rotation acceleration, the algorithm has been mapped to FPGA logic blocks to be executed in the form of groups of engines equipped with our optimized modules, which make full use of the data throughput and computing power of the FPGA. Due to the large data volume, the data coming through PCIe is first cached in the FIFO (First Input First Output). When the data input cycle comes, multiple sets of coordinate and visibility data are obtained from FIFO into the FPGA accelerator module and distributed to the multiple on-call computing kernels for processing. In the remaining steps, the data flow goes through the upgraded customized pipeline to make data available for gridders.

To the best of our knowledge, the phase rotation algorithm has not been implemented in the form of hardware acceleration in previous work, except ours. In this work, for the application scenario of radio astronomical imaging, we proposed an algorithm hardware acceleration method, in which input data are distributed to multiple parallel computing engines whose hardware pipelines are subtly designed to reduce the iteration interval to further improve the throughput of the data flow.

## 6. Gridding Acceleration

### 6.1. Gridding Algorithm Introduction

Gridding in the faceting algorithm is essentially the convolution of the visibility with the convolution kernel, which prompts the two-dimensional convolution to be the dominant operation of the gridding algorithm. The computational formula of each sampled point is as follows:

(6) $$grid\left(u,\;v\right)=\overset{support}{\underset{x=-support}{\sum }}\overset{support}{\underset{y=-support}{\sum }}Samples\left(u-x,v-y\right)*C\left(-x,-y\right)$$

where support refers to the support size of the convolution kernel filter, from which the width of the convolution kernel is thereby derived as (2×support+1). The basic operations applied for visibilities gridded onto a uniform grid is to acquire two pieces of data and to execute a floating-point complex, multiply accumulate operation on them; finally, calculation result is written back to the memory, given that all sampled data and coefficient matrixes are in single-precision floating-point complex format. If the symbol *num_sample* is used to represent the number of samples, we need $num\_sample\times {\left(2\times support+1\right)}^{2}$ computing operations for such samples, which means that the gridding algorithm requires a large amount of computation.

**Figure 10:**
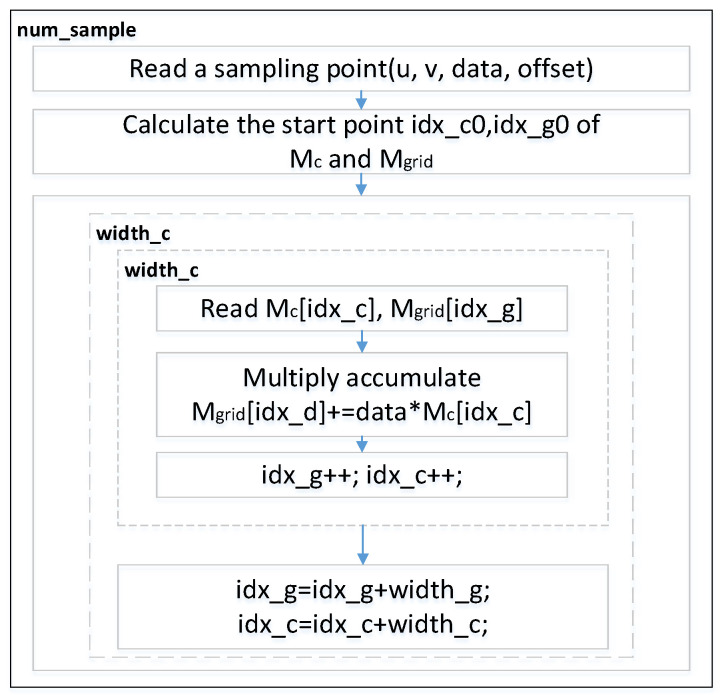
Data flow of the gridding algorithm.

We derive a general gridding execution flow from the standardized measurement program designed for the corresponding algorithm that ASKAP releases, as is shown in Figure 10. The coordinate index lookup table and the convolution coefficient matrix are firstly generated during the preprocessing period. Then, we search for the convolution kernel through sampled data and the map table, then multiply the sampled data with the matching convolution kernel. The computed result is finally affiliated with gridding data. We use *idx_g* and *idx_c*, representing the coordinates of the specific grid and the index of the convolution coefficient matrix, respectively.

### 6.2. Existing FPGA Acceleration System of Gridding

Wu et al. [40] propose an acceleration system applied for the gridding algorithm involved in the hardware implementation. With view to the huge volume of the data to be processed by the SKA scientific data processing application for the gridding algorithm, the work originally completed a design aimed at a small-scale data acceleration system. According to the regular measurement approach that ASKAP exploited, the imported data into the gridder is divided into two sets. One group represents the sampled data of which each is split into three variables, namely *iu*, *iv*, and *data*, among which *iu* and *iv* respectively, represent the location information of the sampling points on two distinct dimensions and the variable of data refers to the spectral value. The other set is a convolution coefficient matrix, *M_c_*, whose width is *width_c*. It is also produced by preprocessing. The running result is the form of a two-dimensional matrix with the size of (width_g×width_g). The details of key data scale can be seen in Table 3. 

The processing flow of Wu’s gridding method [40] is as follows:

1. Import one piece of the sampled point data from the memory
2. Gain the starting address of the region involved in the covered grids through calculation
3. Gain the address of the coefficient matrix of the corresponding sampled data
4. Import the grid matrix data and coefficient matrix data from the memory
5. Overlay the product of the coefficient matrix and the sample data to the grid point data at the current address through the addition operation
6. Write the calculation result in a memory location

It takes 40,021 computation operation cycles for the testbench to execute such procedures of the pipeline structure in an actual hardware test environment, with only 27,300 cycles needed for the same measurement process in the optimal case, theoretically. The efficiency is only 68.2%, though the acceleration ratio can reach 5.2 compared to the software testbench on CPU.

### 6.3. Optimization of FPGA Acceleration System for Gridding

Given that data hazards occur, when executing procedures that exhibit data dependence modifies the data in different stages of a pipeline, we give a detailed analysis of the data conflicts that data hazards induce since the conflict mainly concerns the efficiency of the pipeline. When the supporting area of the currently sampled point is overlapped by that of the last adjacent point, data conflicts occur on the grids involved in the intersection where the previously sampled point data has not been written back into the memory location. It will result in an error if the next point reads the grid data in the scope from BRAM. Therefore, it is required that the address of the current point is compared to that of the last point to determine if an intersection exists. If overlap arises, the data of the point in process is dumped to the beforehand allocated buffer and the next point is delivered into the judgment procedure, otherwise the current point is reasonably passed on the pipeline structure. After each interval, the sample point data is sent as priority to determine the conflict. If the buffer is empty, what follows is the fresh point from the memory. When the buffer fails to hold more sampled points, a point with null value serving as a bubble is inserted into the pipeline to avoid a stall. Imprecisely, the overlap judgment criterion will contribute to an excess of bubbles which aggravates the stagnation of the pipeline, or even an error. Due to the insufficient precision of Wu’s judgment method of the overlapped grids, the number of bubbles exceeds that of conflicts to reduce efficiency.

**Figure 11:**
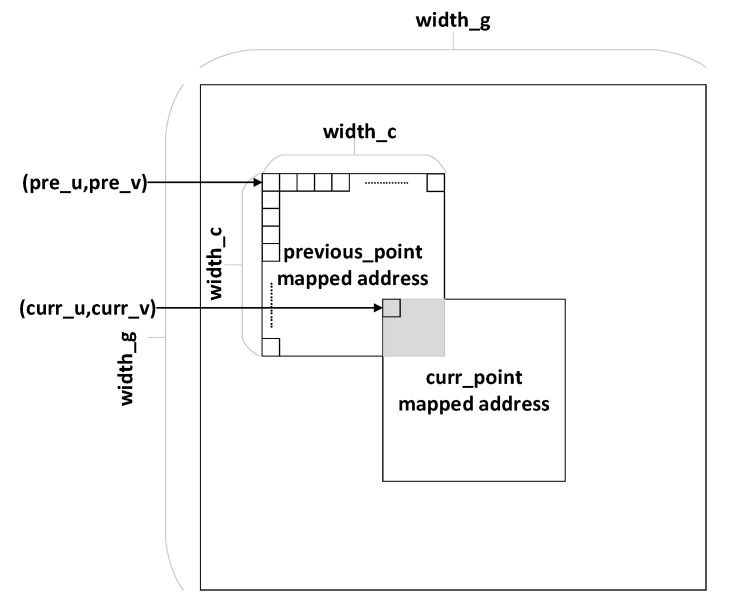
Grid data conflict condition.

The gridding data and sampled point data are serialized by a one-dimensional linear storage hierarchy in terms of the storage model. In contrast, the data model of grid points obeys a two-dimensional structure regarding logic coordinate system and agreed data units, which is shown in Figure 11.

Given that the value of each gridded point is determined by the sampled data of the adjacent scope, the mapped grid matrix is outputted in the form of a two-dimensional matrix of *width_g* × *width_g*. The scope of grids affected by one sampled point depends on the size of the convolution kernel organized as a matrix of *width_c* × *width_c*. The overlapped supporting area of two sampled points is not allowed to prevent the gridded points from being accessed through the current sampled point in process before being updated completely by the previous flow of the adjacent point, due to the identical gridded data still being indeterminate and unavailable. Therefore, only when curr_u∈[pre_u−width_c,pre_u+width_c] and curr_v∈[pre_v−width_c,pre_v+width_c] are both satisfied will the addressed conflict among the two samples emerge. The optimized judgment method enhances the precision of conflict verdicts and avoids unnecessary bubble insertion to reduce the pipeline stagnation.

## 7. Experiment Results and Analysis

After the theoretical analysis of the key algorithm of the facet and FPGA-based implementation of the algorithm, this section concentrates on validating the data processing optimization performance through simulations and experiments on different platforms. The experiment results confirm that the work not only shows the feasibility of the approach of the algorithm accelerated on a specific extended hardware platform, but also achieves significant improvement through FPGA implementation.

The CASA has effectively implemented faceting algorithms covering phase rotation and gridding. We use the software implemented based on the CASA core in which the algorithm is described by C/C++ and is wrapped with Python interface for access. The software baseline based on low-level language typically gets the utmost of the CPU performance. Therefore, we choose the CASA core as the software baseline. Considering that HDL (Hardware Description Language) is superior to high-level synthesis language in performance, we choose Verilog as the FPGA developing language.

In order to realize the real results measured on the FPGA board, simulation is combined with ILA (Integrated Logic Analyzer) to evaluate the running effect. The customizable ILA IP core is a logic analyzer core that can be used to monitor the internal signals of a design. The ILA core includes many advanced features of modern logic analyzers on the FPGA board and signal view display, such that we can derive the performance that is almost as good as it is.

### 7.1. Evaluation of Phase Rotation Optimization

Although current work is aimed at the evaluation on the hardware acceleration effect of phase rotation at this stage, considering that the implementation of remaining procedures on FPGA still need to consume a lot of resources, we chose the accelerator with abundant resources on the chip as far as possible. The Xilinx FPGA provided a stable developing environment and running environment as the dominating vendor whose design compatibility would be the best among all the vendors. The target platform we selected is Xilinx Virtex UltraScale + VCU1525 FPGA, where I/O resource and block RAM are sufficient and a 16-lane PCIe connector is implemented which performs data transfer at the rate of 8.0 GigaTransfers per second at maximum [41], since multi-engines designs need support of high data input bandwidth.

In this work, we unify the procedures including the hardware development, deployment, and test simulation into the Vivado 2019.2 platform that Xilinx corporation provides. In the measurement work, a module applied for data serialization is attached to the test program to transfer the visibility generated during the phase rotation period from the memory to a data flow file stored as a .dat file. The testbench of ARL is executed on a platform with a CPU, namely Intel Core i5-8400, with a basic frequency of 2.8 GHz.

The comparison of the phase rotation acceleration effect among different methods is shown in Table 4 [9]. There are 95,865 sampled data processed in a phase rotation test. The FPGA-based DKs method completes the test in 1.721 × 10^9^ operation cycles, which reaches an acceleration rate 32.07 times higher compared to that on CPU. After the pipeline is fully optimized in the one computing kernel, our novel approach can achieve the same amount of sampling points processed in 9.59 × 10^4^ operation cycles, 20 times higher throughput compared with the DKs.

We compare the phase rotation running result on the hardware platforms to the result obtained by the mathematical software MATLAB. The magnitude order of the absolute error calculated by the difference between the actual value and the measured value is figured out to be 10^−38^, with a maximum relative error of only 4.5 × 10^−5^, calculated by the ratio of absolute error to the measured value. The relative error meets the constraint condition, namely less than 10^−4^, which verifies the functional correctness of the phase rotation acceleration system based on the hardware.

The general FPGA-based accelerator structure of phase rotation is given in Section 5.4, with critical parameters to be determined. The rationalized configuration can fully utilize on-chip resources to optimize hardware structure. Therefore, in order to maximize the throughput of the raw data delivered into the system, the data bandwidth of the buffered queue is required to be taken up. The input throughput can reach 12.8 GB/s in a working frequency of 100 MHz, on the condition of a maximum FIFO bandwidth up to 1 kbit/s.

Given the circumstances of the confined input data, one group of data composed of a coordinate of three dimensions and a piece of visibility, each sample will make up (32 × 5) bits width, which is followed by a single computing kernel to be processed. Thus, the maximum amount of the kernels is calculated to be 6, which indicates that there is a maximum of 6 kernels in parallel to process the data from the FIFO, theoretically. However, the number of kernels is bound to be limited by resources on-chip of the specific FPGA platform. Table 5 shows the details about resource utilization for the FPGA-based algorithm accelerator. The test result indicates that the FPGA we selected provides sufficient on-chip resources to support multiple kernels, up to 6, to achieve complete phase rotation acceleration in parallel. Faced with a large volume of data in SKA, our method based on FPGA can support 2 GB/s imaging data processed by each engine in the multi-engine design, which outperforms the existing methods. Transmission bandwidth becomes a dominating performance bottleneck, and as the device is constantly updated, throughput can be continuously improved through our approach.

### 7.2. Evaluation of Gridding Optimization

In order to evaluate the efficiency of gridding optimization, we used Vivado 2019.2 for simulation tests. The referenced software test program is operated on a system with a CPU type 2x Intel Xeon E5-2620 v4.

The performance is shown in Table 6. As the simulation result shows, the optimized system completes the gridding algorithm with 1820 sampling points in 38,000 operation cycles, and acceleration rate access is 5.48 compared to the CPU software running result. The calculation efficiency comes up to 71.9% and has a 5% increase in performance compared to Wu’s work. But, the pipeline stagnation still exists. To eliminate it more thoroughly, it is an appropriate choice to increase the buffer depth, which not only reduces the occupation of hardware source but improves the performance. Table 7 shows the details about resource utilization for the FPGA-based algorithm accelerator.

We compared the gridding running result on the hardware platforms to the result obtained by MATLAB. The magnitude order of the absolute error is figured out to be 10^−38^ with a maximum relative error of only 4.5 × 10^−5^. The relative error meets the constraint condition, namely less than 10^−4^, which verifies the functional correctness of the gridding acceleration system based on the hardware.

In view of the original single precision floating point data, FPGA can use DSP48 to complete single precision floating point calculations. In our approach, lots of DSPs are used in the gridding part. For the other parts without DSP used, we focused on the pipeline optimization to achieve high throughput and low computational delay. In order to reduce the computational complexity of FPGA, we designed a module of transformation from the floating-point to the fixed-point for subsequent processing. The experimental results indicate that the precision requirement is satisfied. In the future, we will consider using a variable precision calculation to better customize the calculation of astronomical big data.

### 7.3. Energy Efficiency Comparison of Distinct Hardware Implementation

In consideration of the read/write-intensive application that faceting is, the target platforms should have the approximate theoretical bandwidth of off-chip memory to guarantee the fair comparison among distinct hardware architecture. The DDR theoretical bandwidth of the VCU1525 FPGA board is about 76.8 GB/s, and the GDDR theoretical bandwidth of the GeForce GTX650 GPU is approximately 80 GB/s. With respect to the performance evaluation, SPPS (Sampling Points Per Second) in faceting procedures is more intuitive than other metrics. The running energy efficiency comparison of faceting on different platforms is shown in Table 8.

The GPU results of the CUDA benchmark are approximately 4.45× better than the CPU benchmark. The performance of the FPGA-based implementation is a bit superior to the GPU benchmark. In the scenarios of astronomical observation, the power dissipation of the devices cannot be ignored. Thus, the energy efficiency of faceting on different hardware architecture should be considered. To eliminate the effect of the power of the units in standby mode on different platforms, we measured the running power of each platform (the power difference of the target platform with and without the application running) to reveal the power dissipation by a digital power meter. Firstly, we measured the static power of each platform without the target application at runtime and then measured the dynamic power with the target application (CPU, CUDA, FPGA) running. The power at runtime is the difference of dynamic and static power. The power of each platform is separately measured 10 times and averaged to generate the numerical result. EyE (Energy Efficiency) is calculated from SPPS and power, which is shown as follows:

(7) EyE=Top/Power

The running power and energy efficiency of faceting on different hardware are shown in Table 8. The FPGA-based prototype achieves 15.57 times and 4.33 times more energy efficiency than the software benchmark and CUDA-based benchmark on the target CPU and GPU, respectively.

## 8. Conclusions

To handle the challenges of insufficient computational performance for the big data processing produced by the largest sensor arrays in the world, we proposed a complete accelerator design for a major imaging algorithm, faceting, which was not explored for complete acceleration on FPGA before. Targeting the most time-consuming procedures in the faceting, we discussed a feasible FPGA-based acceleration system for them and proposed an efficient parallelization and pipeline structure designed for the two algorithms and implemented them on the FPGA. The test result shows that the preliminary acceleration ratio on the FPGA of the phase rotation algorithm is 32.07 and gridding is 5.48, compared to that of software running on the CPU, and the acceleration ratio optimized accelerator of phase rotation has a 20 times higher performance compared with the previous design on the FPGA. Besides, the FPGA-based implementation outperforms CPU and GPU in terms of energy efficiency.

In this work, we set out to implement the key procedures of the faceting algorithm based on FPGA, which illustrates the feasibility of the optimization scheme. The method we presented in the paper can be alternatively applied to other hardware platforms with higher performance and achieve more optimal computational performance. Besides, through the research of processing acceleration of radio astronomical imaging data, the extraordinary approaches to handle the challenge of processing primarily oriented toward massive data flow can be extended to other application scenarios of sensor arrays.

## Figures and Tables

**Table 1:** Time consumed in various stages.

Stage	Time (s)
Freq (Frequency Averaging)	0.012 s
FFT (Fast Fourier Transform)	0.0007 s
Phase Rotation	0.6148 s
Gridding	2.5693 s

**Table 2:** Data magnitude in phase rotation acceleration system.

Data	Data Type	Number
Visibility	Single-precision floating-point complex	95,865
Coordinate (u, v, w)	Single-precision floating-point	95,865
l, m	Single-precision floating-point	1

**Table 3:** Data characteristics in small-scale acceleration system.

Sampling Numbers (*Num_Sample*)	1820
Width of grid (*width_g*)	128
Length of convolution coefficient matrix	1800
Support size of convolution kernel (*support*)	7
Width of convolution kernel (*width_c*)	15

**Table 4:** Comparison of phase rotation acceleration system and software test.

	Standardized Test Software	Design of DKs	Design of Optimized Structure
Platform	Intel Core i5-8400	Xilinx VCU1525	Xilinx VCU1525
Number of sampling points	95,865	95,865	95,865
Clock frequency	2.8 GHz	100 MHz	100 MHz
Operation cycles	1.721 × 10^9^	1.917 × 10^6^	9.59 × 10^4^
Time consumed per sampling point (ns)	6413.2	139.2	6.96

**Table 5:** Resource utilization report of phase rotation.

Site Type	Used	Available	Util%
CLB	721	27,120	2.66
LUT as Logic	2496	216,960	1.15
LUT as Memory	429	99,840	0.43
CLB Registers	4009	433,920	0.92
Unique Control Sets	213	54,240	0.39
Block RAM Tile	1	480	0.21
GLOBAL CLOCK BUFFERs	5	256	1.95

**Table 6:** Comparisonof gridding acceleration ratio.

	Standardized Test Software	Simulation Result
**Number of sampling points**	1820	1820
**Clock frequency**	2.1 GHz	150 MHz
**Operation cycles**	3.624 × 10^8^	38,006
**Time consumed per sampling point (ns)**	761	139.2

**Table 7:** Resource utilization report of gridding.

Site Type	Used	Available	Util%
LUT	20,836	433,200	4.81
LUT RAM	435	174,200	0.25
FF	13,296	866,400	1.53
BRAM	238	1470	16.19
DSP	60	3600	1.67
IO	144	600	24.00

**Table 8:** Performance (MPoints/s, million sampling points per second), running power (Watt), and running energy efficiency (MPoints/J) of faceting on different platforms.

Platform	Performance	Running Power	EyE (Top/Power)
Intel Core i5-8400	1.314	37.41	0.035
GeForce GTX650	5.859	46.67	0.126
Virtex UltraScale + VCU1525	7.194	13.21	0.545

## References

[1] Schilizzi R.T. The square kilometer array. Proceedings of the Ground-based Telescopes.

[2] Turner W., Cornwell T., McPherson A., Diamond P. (2014). Ska Phase 1 System (Level 1) Requirements Specification.

[3] Stergiopoulou A. (2016). Combining E-ELT HIRES Instrument and SKA to Probe the Chemical Enrichment by the First Stars. Ph.D. Thesis.

[4] Dewdney P.E., Turner W., Millenaar R., McCool R., Lazio J., Cornwell T.J. SKA1 system baseline design. SKA-TELSKO-0000002 Rev 2016; 3. https://www.skatelescope.org/wp-content/uploads/2013/03/SKA-TEL-SKO-DD-001-1_BaselineDesign1.pdf.

[5] Kogan L., Greisen E.W. (2009). Faceted Imaging in AIPS. AIPS Memo.

[6] Tayara H., Ham W., Chong K.T. (2016). A Real-Time Marker-Based Visual Sensor Based on a FPGA and a Soft Core Processor. Sensors.

[7] Huang J., Zhou G., Zhou X., Zhang R. (2018). A New FPGA Architecture of FAST and BRIEF Algorithm for On-Board Corner Detection and Matching. Sensors.

[8] Jongerius R., Wijnholds S., Nijboer R., Corporaal H. (2014). An end-to-end computing model for the square kilometre array. Computer.

[9] Nan T., Zhu Y., Li W., Chen X., Song Y., Hou J. An FPGA-based Hardware Acceleration for Key Steps of Facet Imaging Algorithm. Proceedings of the Smartcloud Meeting.

[10] Veenboer B., Petschow M., Romein J.W. Image-domain gridding on graphics processors. Proceedings of the IEEE International Parallel & Distributed Processing Symposium (IPDPS).

[11] Cornwell T.J., Golap K., Bhatnagar S. Wide field imaging problems in radio astronomy. Proceedings of the IEEE International Conference on Acoustics, Speech, and Signal Processing.

[12] Perley R.A. (1999). Synthesis Imaging in Radio Astronomy II ASP Conference Series.

[13] Tasse C., Hugo B., Mirmont M., Smirnov O., Atemkeng M., Bester L., Hardcastle M.J., Lakhoo R., Perkins S., Shimwell T. (2018). Faceting for direction-dependent spectral deconvolution. Astron. Astrophys..

[14] Cornwell T.J., Golap K., Bhatnagar S. (2008). The Noncoplanar Baselines Effect in Radio Interferometry: The W-Projection Algorithm. IEEE J. Sel. Top. Signal Process..

[15] Humphreys B., Cornwell T. Analysis of Convolutional Resampling Algorithm Performance. https://www.skatelescope.org/uploaded/59116_132_Memo_Humphreys.pdf.

[16] Cornwell T.J., Voronkov M.A., Humphreys B. Wide field imaging for the Square Kilometre Array. Proceedings of the SPIE—The International Society for Optical Engineering.

[17] Johnston S., Bailes M., Bartel N., Baugh C.M., Bietenholz M., Blake C., Braun R., Brown J., Chatterjee S., Darling J. (2007). Science with the Australian Square Kilometre Array Pathfinder. Publ. Astron. Soc. Aust..

[18] SKAO Frequently Asked Questions. https://skatelescope.org/frequently-asked-questions/.

[19] Bolton R., Malan F., Nijboer R., Scaife A., SDP Architecture Group (2015). SDP Meeting at ASTRON Netherland. http://www.astron.nl/~broekema/papers/SDP-PDR/PDR01%20System%20Architecture.pdf.

[20] TOP500 LIST TOP10. https://www.top500.org/lists/2019/06/.

[21] Sabater J., Sánchez-Expósito S., Best P., Garrido J., Verdes-Montenegro L., Lezzi D. (2017). Calibration of LOFAR data on the cloud. Astron. Comput..

[22] Wang Y., Yang J., Guo X., Qu Z. (2019). Satellite edge computing for the internet of things in aerospace. Sensors.

[23] Marinescu D.C. (2013). Chapter 11—Cloud application development. Cloud Comput..

[24] Humphreys B., Cornwell T. (2001). Analysis of convolutional resampling algorithm performance. SKA Memo.

[25] Romein J.W. An efficient work-distribution strategy for gridding radio telescope data on GPUs. Proceedings of the 26th ACM International Conference on Supercomputing (ICS).

[26] Merry B. (2016). Faster GPU-based convolutional gridding via thread coarsening. Astron. Comput..

[27] Lao B., Tao A., Yu A., Guo S. (2019). Research on parallel algorithms for uv-faceting Imaging. Chin. Astron. Astrophys..

[28] Veenboer B., Romein J.W. Radio-astronomical Imaging: FPGAs vs GPUs. Proceedings of the European Conference on Parallel Processing.

[29] Li J., Ming Z., Qiu M., Quan G., Qin X., Chen T. (2011). Resource allocation robustness in multi-core embedded systems with inaccurate information. J. Syst. Archit..

[30] Li J., Qiu M., Niu J., Gao W., Zong Z., Qin X. Feedback Dynamic Algorithms for Preemptable Job Scheduling in Cloud Systems. Proceedings of the IEEE/WIC/ACM International Conference on Web Intelligence & Intelligent Agent Technology IEEE Computer Society.

[31] Vestias M., Neto H. Trends of CPU, GPU and FPGA for high-performance computing. Proceedings of the 2014 24th International Conference on Field Programmable Logic and Applications (FPL) IEEE.

[32] Perley R.A., Taylor G.B., Carilli C.L., Perley R.A. (1999). Imaging in Radio Astronomy II. A Collection of Lectures from the Sixth NRAO/NMIMT Synthesis Imaging Summer School, ASP Conference Series.

[33] Muscat D. (2014). High-Performance Image Synthesis for Radio Interferometry. arXiv.

[34] Lao B.-Q., An T., Chen X., Wu X.C., Lu Y. (2017). Research on Wide-field Imaging Technologies for Low-frequency Radio Array. Acta Astron. Sin..

[35] Iupikov O.A., Ivashina M.V., Smirnov O.M. Reducing the complexity of the beam calibration models of phased-array radio telescopes. Proceedings of the 5th European Conference on IEEE Antennas and Propagation (EUCAP).

[36] SKA-ScienceDataProcessor Algorithm-Reference Library. https://github.com/SKA-ScienceDataProcessor/algorithm-reference-library.

[37] Meher P.K., Park S.Y. (2019). Design of Cascaded CORDIC Based on Precise Analysis of Critical Path. Electronics.

[38] Fan Y.-C., Liu Y.-C., Chu C.-A. (2019). Efficient CORDIC Iteration Design of LiDAR Sensors’ Point-Cloud Map Reconstruction Technology. Sensors.

[39] Qiao W., Du J., Fang Z., Wang L., Lo M., Chang M.-C.F., Cong J. High-Throughput Lossless Compression on Tightly Coupled CPU-FPGA Platforms. Proceedings of the 2018 IEEE 26th Annual International Symposium on Field-Programmable Custom Computing Machines (FCCM).

[40] Wu Q., Zhu Y., Wang X., Li M., Masoumi A. Exploring High Efficiency Hardware Accelerator for the Key Algorithm of Square Kilometer Array Telescope Data Processing. Proceedings of the 2017 IEEE 25th Annual International Symposium on Field-Programmable Custom Computing Machines (FCCM).

[41] Kavianipour H., Muschter S., Bohm C. (2012). High Performance FPGA-Based DMA Interface for PCIe. IEEE Trans. Nuclear Sci..

